# A comparison of the life-history traits between diapause and direct development individuals in the cotton bollworm, *Helicoverpa armigera*

**DOI:** 10.1093/jis/14.1.19

**Published:** 2014-01-01

**Authors:** Chao Chen, Qin-Wen Xia, Hai-Jun Xiao, Liang Xiao, Fang-Sen Xue

**Affiliations:** 1 Institute of Entomology, Jiangxi Agricultural University, Nanchang 330045, China; 2 Key Laboratory of Crop Physiology, Ecology and Genetic Breeding, Jiangxi Province,China; 3 These authors contributed equally to this work.

**Keywords:** developmental pathways, protogyny, theremal reaction

## Abstract

In order to understand the differences of life-history traits between diapause and direct development individuals in the cotton bollworm, *Helicoverpa armigera* (Hübner) (Lepidoptera: Noctuidae), the development time, body size, growth rate, and adult longevity were investigated between the two populations, which were induced under 12:12 L:D and 16:8 L:D photoperiods, respectively, at 20, 22, and 25°C. The results indicated that the larval development time, pupal weight, adult weight, and growth rate were significantly different between diapause and direct developing individuals. The diapause developing individuals had a significantly higher pupal and adult weight and a longer larval time compared with direct developing individuals. However, the growth rate in diapause developing individuals was lower than that in the direct developing individuals. Analysis by GLM showed that larval time, pupal and adult weight, and growth rate were significantly influenced by both temperature and developmental pathway. The pupal and adult weights were greater in males than females in both developmental pathways, exhibiting sexual size dimorphism. The dimorphism in adult weight was more pronounced than in pupal weight because female pupae lost more weight at metamorphosis compared to male pupae. Protogyny was observed in both developmental pathways. However, the protogyny phenomenon was more pronounced at lower temperatures in direct developing individuals, whereas it was more pronounced in diapause developing individuals when they experienced higher temperatures in their larval stage and partial pupal period. The adult longevity of diapause developing individuals was significantly longer than that of direct developing individuals. The results reveal that the life-history strategy was different between diapause and direct developing individuals.

## Introduction

Body size, development time, and growth rate are three closely-interrelated life-history traits in insects. These traits are typical evaluation indicators in fitness and they also correlate with other key life-history traits ( Nylin and Gotthard 1998 ). Body size can directly influence competition, longevity, reproduction, and adult migration ( Peters 1986 ; Stearns 1992 ; Honěk 1993 ; Nylin and Gotthard 1998 ; Roff 2002 ). Small individuals may survive and reproduce better when food is limited because they need less food to sustain themselves ( Dingle 1992 ; Blanckenhorn et al. 1995 ). On the other hand, larger individuals may survive better when there is no food at all, e.g., during hibernation, if body size is correlated with nutrient reserves ( Ohgushi 1996 ). In general, a short development time is beneficial in non-seasonal environments because it reduces the risk of death before reproduction ( Sibly and Calow 1986 ); however a long development time may lead to a larger body size, which is also important to fitness ( Roff 1992 ; Stearns 1992 ). In seasonal environments, development time might influence potential reproductive success ( Wickman 1992 ), host plant utilization ( Yela and Herrera 1993 ), and escaping from predation ( McGregor 1996 ). In most situations, a high growth rate is beneficial. In accordance with this, ovipositing females of phytophagous insects typically show a preference for host plants capable of supporting fast larval growth ( Thompson 1988 ; Janz et al. 1994 ).

In many insect species, body size, development time, and growth rate are sexual dimorphisms. The sexual dimorphism in body size is called sexual size dimorphism and females are usually larger than males. The sexual size dimorphism may be a result of different selection pressure between sexes ( Masaki 1967 ; Landman et al. 1989 ; Mousseau and Roff 1989 ; Roff and Mousseau 2005 ; Berner and Blanckenhorn 2006 ; Bidau and Martí 2007 ). The development time in insect species is usually asynchronous between sexes and is called sexual development time dimorphism. Protandry is commonly observed in insect species, such as in most butterflies and moths, but protogyny is rarely observed ( Thornhill and Alcock 1983 ).

Within species, body size, development time, and growth rate commonly are different between diapausing and direct developing individuals ( Nylin and Gotthard 1998 ; Fischer and Fiedler 2001 ). For example, when the butterfly *Pieris napi* entered a pupal diapause in response to short-day conditions it had greater pupal weight, longer larval time, and lower growth rate compared with direct developing individuals ( Nylin and Gotthard 1998 ). In the butterfly *Lycaena tityrus* , which enters larval diapause in the 3 ^rd^ stadium, the comparison of life-history traits between the two developmental pathways was similar to *P. napi* ( Fischer and Fiedler 2001 ).

The cotton bollworm, *Helicoverpa armigera* (Hübner) (Lepidoptera: Noctuidae), is one of the most important agricultural pests and displays a facultative pupal diapause in response to short-day conditions. In recent years, it was found that this moth was a protogynous species, and the difference of emergence was due to a shorter pupal duration in females ( Chen et al. 2012 ).

In the present study, in order to understand the differences of life-history strategies between direct and diapausing developers, body size, development time, growth rate, and adult longevity of diapausing and direct-developing individuals were measured, and the effects of developmental pathway, sex, temperature, and interaction terms on life-history traits were analyzed.

## Materials and Methods

### Study organism

*Helicoverpa armigera* is a widespread insect species, ranging from almost northernmost to southernmost China. The populations of *H. armigera* can be divided into four regional groups (tropical, subtropical, temperate, and Xinjiang geotypes) that have genetic, diapause, and cold-hardiness variations ( Wu and Guo 2007 ). The *H. armigera* for this study were collected from Langfang city (39° 31’ N, 116° 42’ E), Hebei Province, and belonged to the temperate geotype. In this region, *H. armigera* produces two or three generations per year and harms crops from early June to late September. Dozens of full-grown larvae were caught in cornfields in July 2010. The full-grown larvae were reared on an artificial diet ( Wu and Gong 1997 ) in plastic plates with 21 holes (for each hole: length: 2.5 cm; width: 2.5cm; height: 2.5 cm) and kept under a 16:8 L:D photoperiod at 25 ± 1° C until pupation. When the larvae pupated, they were individually placed in new plastic plates for eclosion. On the day that adults emerged, the adults were fed with 10% honey solution and mated in a group of about 50 pairs in a 40 ×25 ×18 cm cage with a removable gauze cloth top for egg collection. Eggs were maintained under a 16:8 L:D photoperiod at 25 ± 1° C until hatching. After hatching, every three to five newly-hatched larvae were transferred together to the plastic plates with 24 holes (for each hole: diameter: 1.5 cm; height: 2 cm) and reared on an artificial diet until the 3 ^rd^ stadium, and then larvae were reared individually in plastic plates with 21 holes until pupation. On the day that adults emerged, adults were sexed and eggs were collected as mentioned above. The eggs that were produced in the first two days were used to carry out the experiments.

### Experimental methods

After hatching, the newly hatched larvae were randomly divided into different groups, some of which were exposed to a constant short photoperiod of 12:12 L:D at 20, 22 and 25° C to induce diapause; the incidence of pupal diapause (the eyespots did not move after pupating for 10 to 15 days ( Cullen and Browning 1978 )) was 95.7% at 20° C, 88.1% at 22° C and 68.7% at 25° C. The other larvae were exposed to a long photoperiod of 16:8 L:D at 20, 22 and 25° C to induce direct development; all individuals pupated without diapause at all temperatures. The development time of all treatments from hatching to pupation were recorded in detail. The pupae were weighed on the second day after pupation. In this experiment, the data of non-diapausing individuals produced under short-day conditions were not used, because the larval duration and pupal weight for non-diapausing individuals were between the direct developing individuals and diapausing individuals.

To examine whether there was a difference in adult weight between the diapausing and direct developing individuals, developing pupae exposed to long-day conditions were kept at their larval rearing temperatures until adult eclosion. Diapausing pupae exposed to short-day conditions were kept at their larval rearing temperatures for 10 days at 25° C, 12 days at 22° C and 15 days at 20° C to ensure pupae being in diapause, and then they were moved to 25° C and 16:8 L:D to terminate diapause. Thus, the pupal stage of diapausing individuals included the prediapause, diapause development and postdiapause periods. Adults were weighed on the day of eclosion after having excreted meconium. The electric balance used was the AUY120 produced by SHIMADZU ( www.shimadzu.com ).

To estimate whether diapause developing individuals have shorter adult life spans than direct developing individuals, both the newly emerged adults produced under a short photoperiod (12:12 L:D at 20° C) and the ones produced under a long photoperiod (16:8 L:D at 20° C) were placed under a 16:8 L:D photoperiod at 25° C to observe their longevity. In this experiment, diapausing individuals experienced 76 days from hatching to adult (45 days at 20° C, 31 days at 25° C for diapause termination), whereas direct developing individuals experienced 49 days from hatching to adult.

From the above data, the relative growth rate of direct and diapausing developing individuals was calculated according to Gotthard et al. (1994) :

$\text{Growth rate} = [\text{ln} (\text{pupal weight}) - \text{ln} (\text{hatching weight})] / \text{larval time} \times 100\%$

The growth rate of each larvae used in the experiments was calculated as mean weight gain per day. To assess the weight of newly hatched larvae, four samples of 100 larvae each were weighed soon after hatching, their weight being 0.056 ± 0.004 mg on average. Because of the low weight, larvae could only be weighed as groups. Proportional weight loss at metamorphosis was measured using the formula given in Gotthard et al. (1994) :

$\text{Proportion weight loss} = 1 - (\text{adult weight} / \text{pupal weight}).$

### Statistics

Statistical analyses were conducted using the SPSS 17.0 (IBM, www.ibm.com ). Differences of life-history traits among groups were localized using Tukey’s HSD post-hoc comparison at the 5% level after PROC GLM with temperature, sex, and developmental pathway as factors. A comparison of adult longevity between developmental pathways was performed using Kaplan-Meier estimation and log-rank tests. Throughout the text, all means are given ± 1 SE.

## Results

### Larval development time

**Figure 1.**
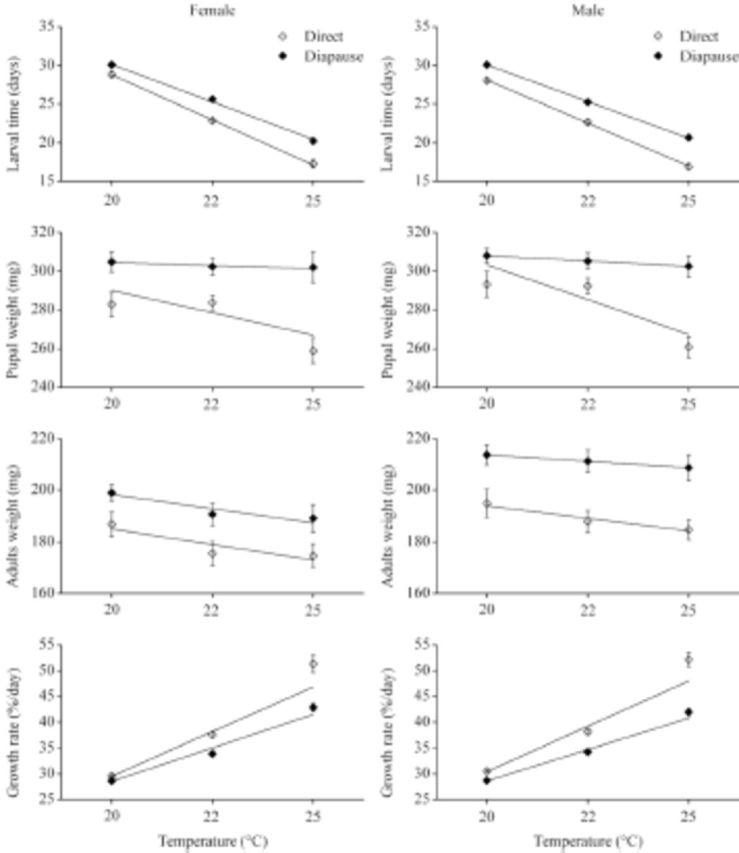
The comparisons of larval time, pupal weight, adult weight, and growth rate between diapause and direct developing individuals of *Helicoverpa armigera* induced at 20, 22 and 25° C (n= 39–70). High quality figures are available online.

Larval development time in diapause development was significantly longer than that in direct development at all rearing temperatures ( Table 1 , 2 ). The extent of the difference between two developmental pathways was significantly influenced by temperature ( Figure 1 ; Table 1 , 2 ).

**Table 1.**
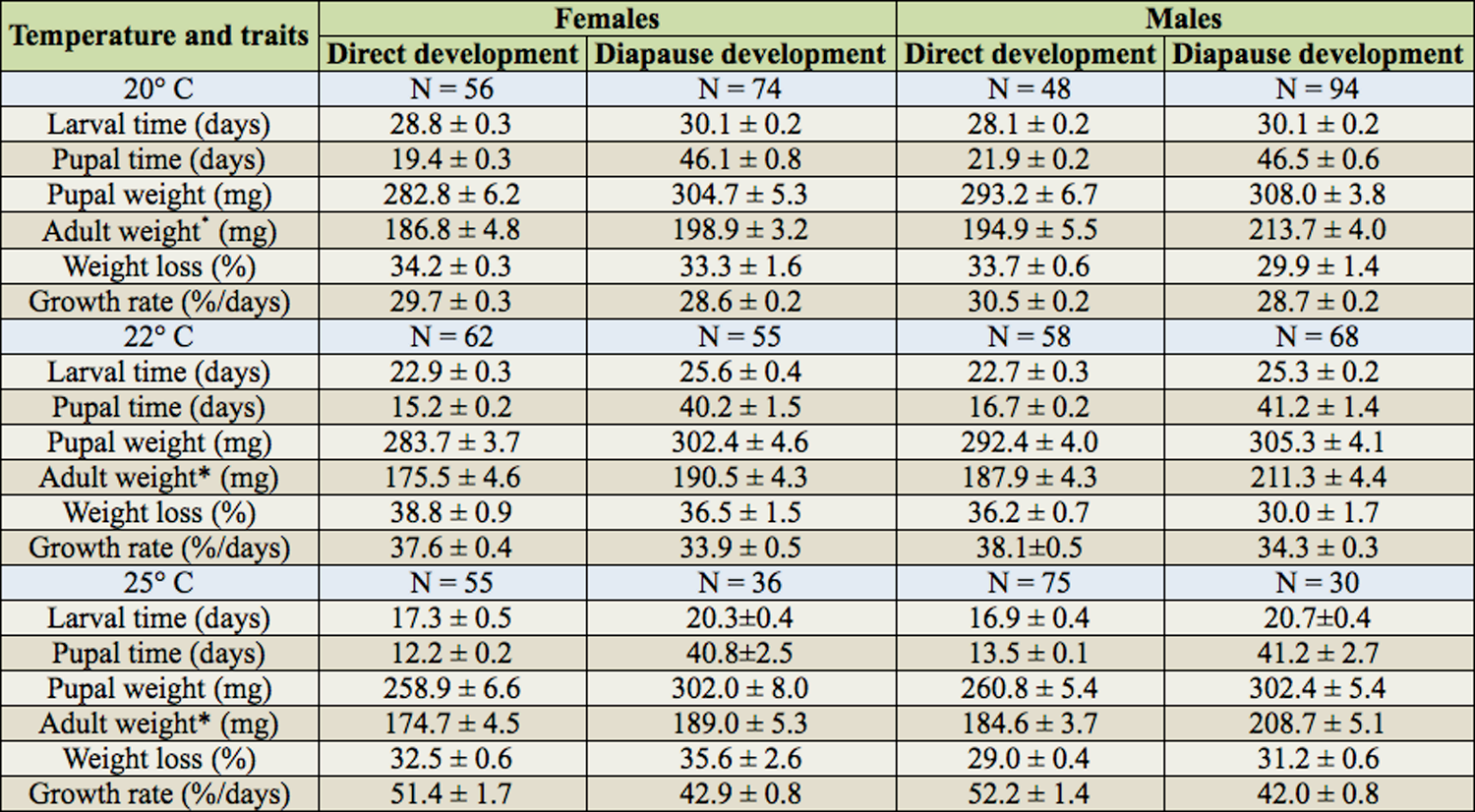
Life-history data (mean ± 1 SE) for male and female *Helicoverpa armigera* compared between direct and diapause development at 20, 22 and 25° C.

**Table 2.**
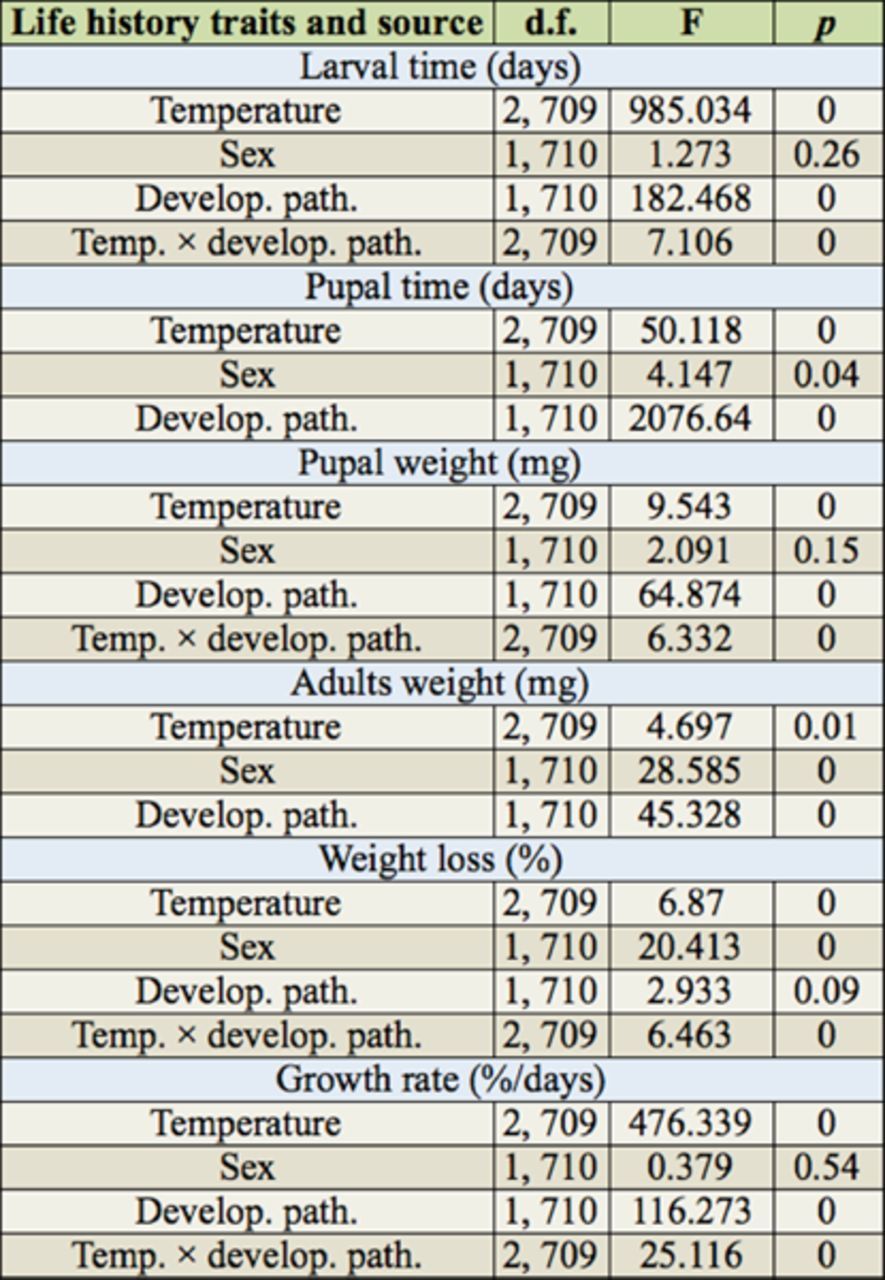
Analysis of fixed effects in life-history traits of *Helicoverpa armigera* in relation to temperature, sex, and developmental pathway by SPSS PROC GLM.

### Pupal and adult weight

**Figure 4.**
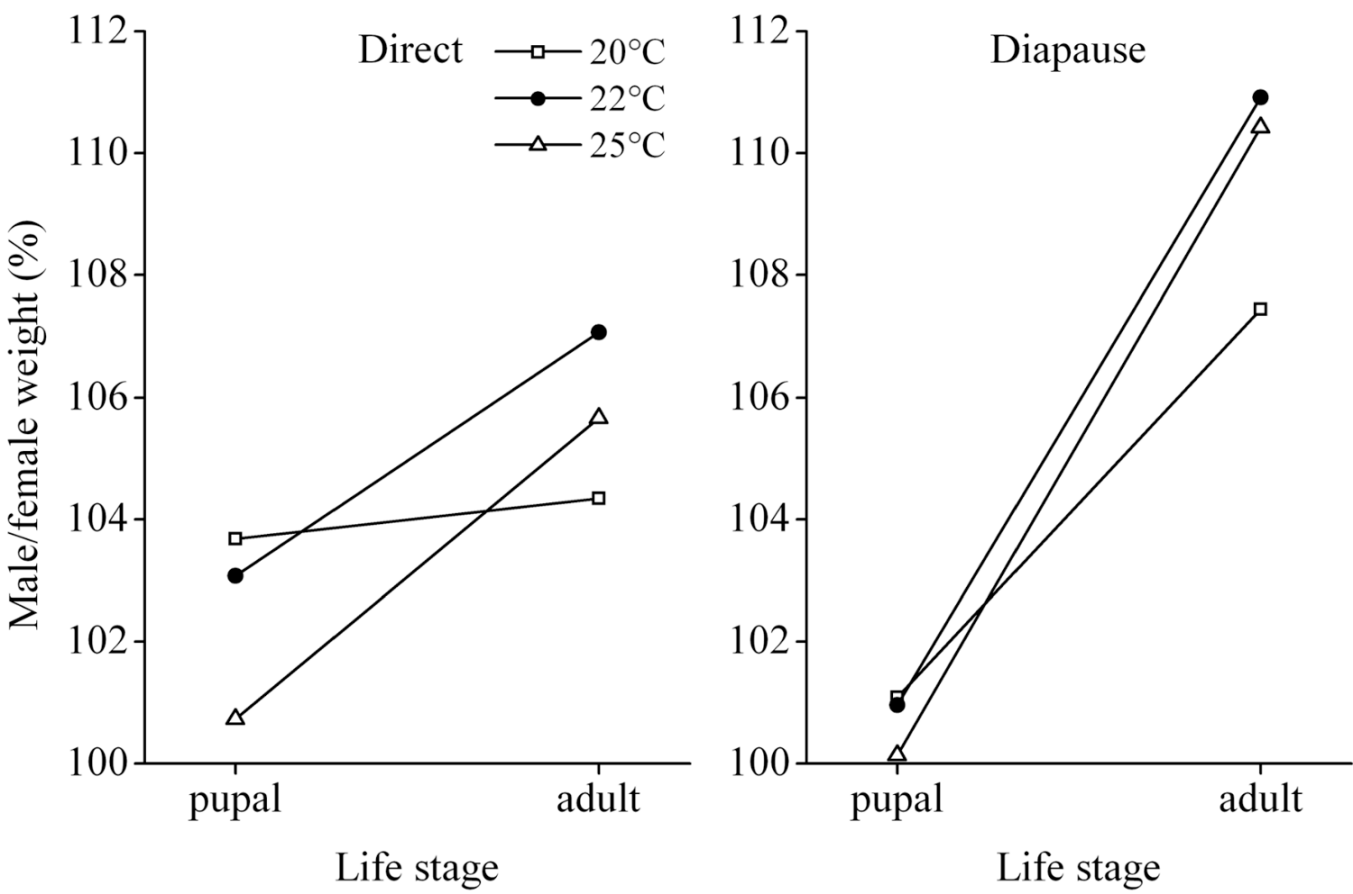
Quotient of male to female pupal and adult weight in direct and diapausing developing *Helicoverpa armigera* under different temperatures. Sexual size dimorphism was more pronounced in the adult stage than at the pupal stage. High quality figures are available online.

Diapause developing individuals reached significantly higher pupal and adult weights than direct developing individuals at all rearing temperatures ( Figure 1 ; Table 1 , 2 ). The pupal weight in direct development was significantly different among temperatures (Tukey’s test: female: F 2, 170 = 6.296, *p* = 0.002; male: F 2, 178 = = 12.660, *p* = 0.000) but not in diapause development (female: F 2, 162 = 0.069, *p* = 0.934; male: F 2, 189 = 0.360, *p* = 0.698). The pupal and adult weights were greater in males than females in both developmental pathways ( Table 1 ). Therefore, sexual size dimorphism appeared in both pupal and adult stages. However, since female pupae lost more weight at metamorphosis compared with male pupae ( Table 1 , 2 ), the dimorphism in adult weight was more pronounced than in pupal weight ( Figure 4 ). The significant sexual size dimorphism only appeared at the adult stage ( Table 2 ).

### Protogyny phenomenon

**Figure 2.**
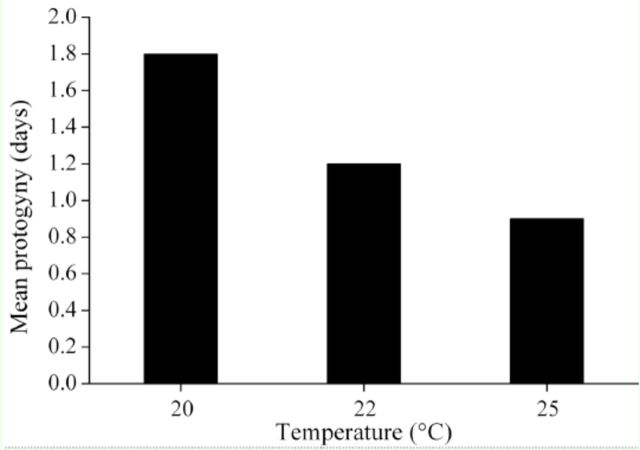
Extent of earlier emergence (protogyny) of females compared to males of *Helicoverpa armigera* . The protogyny that appeared in direct developing individuals was measured as differences between group averages. High quality figures are available online.

**Figure 3.**
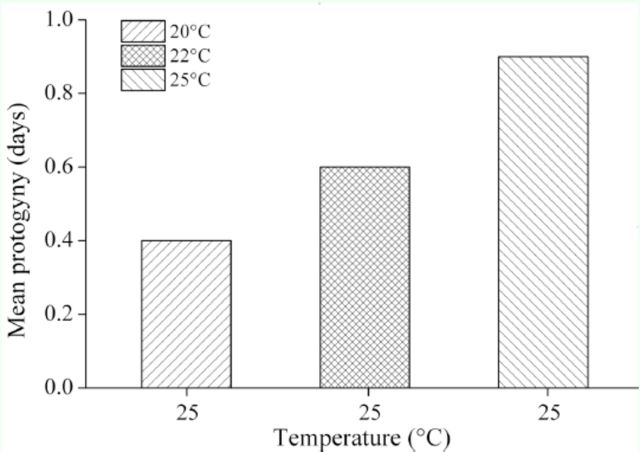
Extent of earlier emergence (protogyny) of females compared to males of *Helicoverpa armigera* . The protogyny that appeared in diapause developing individuals was measured as differences between group averages. High quality figures are available online.

Females in direct development emerged 1.8, 1.2, and 0.9 days earlier than males at 20, 22, and 25° C, respectively ( Figure 2 ). Females in diapause development emerged 0.4, 0.6, and 0.9 days earlier than males at 25° C when their larval stage and partial pupal stage were exposed to 20, 22, and 25° C, respectively ( Figure 3 ), indicating that this moth presents the protogyny phenomenon. Interestingly, the protogyny phenomenon was more pronounced at the low temperature of 20° C than at the high temperature of 25° C in direct development, whereas the protogyny phenomenon was more pronounced in diapause development when the larval stage and partial pupal stage were exposed to the high temperature of 25° C rather than to the low temperature of 20° C. The developmental advantage of females was mainly achieved during pupal development ( Table 2 ), whereas larval time made a lesser contribution, without showing significant differences ( Table 2 ).

### Growth rate

The growth rate was significantly influenced by developmental pathways and temperature, as direct developing individuals had a significantly higher growth rate than diapause ones at all temperatures ( Table 2 ). Temperature significantly influenced the extent of difference in growth rate (temperature ×development pathway interaction: F2, 710 = 25.116, *p* < 0.05; Table 2 ), and the extent increased with increasing temperature ( Figure 1 ).

### Longevity of adults

**Figure 5.**
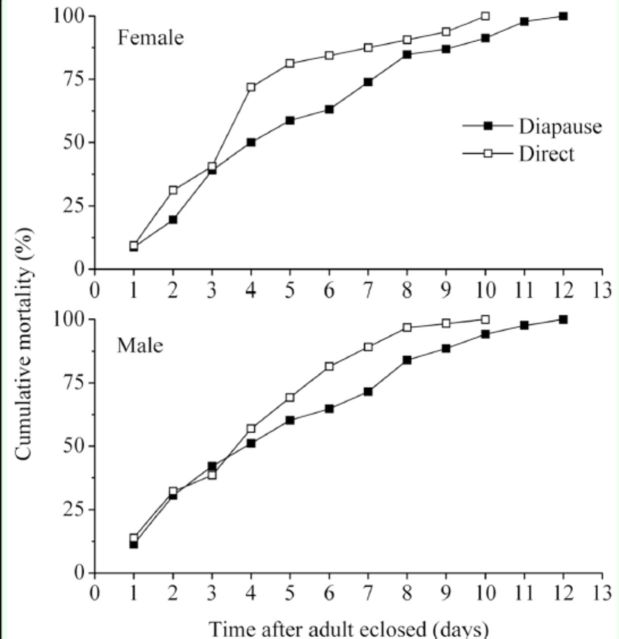
A comparison of longevity of females and males between adults from diapause and direct developing individuals of *Helicoverpa armigera* (n = 47 and 88 for diapausing developing females and males, respectively; n = 32 and 65 for direct developing females and males, respectively). High quality figures are available online.

Figure 5 shows that the cumulative mortality was higher in direct developing individuals than in diapauses developing individuals after the fourth day. Survival analysis showed that the longevity of both female and male adults from diapause developing individuals (5.26 ± 0.45 days for females; 5.03 ± 0.34 days for males) was significantly longer than that from direct developing individuals (4.09 ± 0.43 days for females; 4.23 ± 0.30 for males) (log-rank test: females: χ ^2^ = 3.91, *p* = 0.048; males: χ ^2^ = 5.41, *p* = 0.020).

## Discussion

The results revealed that the life-history traits in this moth were phenotypically plastic in relation to developmental pathway and temperature. The induction of developmental pathway not only affected the diapause phenotype (i.e., whether diapause is expressed or not) but also affected other key life history traits. In accordance with many observations, the direct developmental pathway was associated with a shorter development time ( Wiklund et al. 1991 ; Nylin 1992 ; Blanckenhorn and Fairbairn 1995 ; Wiklund and Friberg 2011 ; Pöykkö and Hyvärinen 2012 ), higher growth rate ( Wiklund et al. 1991 ; Nylin 1992 ; Pöykkö and Hyvärinen 2012 ), and larger body size ( Wiklund et al. 1991 ; Kivelä et al. 2012 ) compared with the diapause pathway. However, the results of our study showed that pupal weight in diapause development was significantly higher than in direct development, especially at the higher temperature of 25° C ( Table 1 ). *H. armigera* overwinters as pupae. Therefore, high body weight in diapausing pupae may be an adaptive strategy. We hypothesize that the diapause individuals need to reach a critical pupal weight to overwinter successfully. Increased body weight may deal with the energy demand of the diapause period and ensure a high fecundity after a long diapause period ( Wei et al. 2010 ). For example, the butterfly *Lycaena hippothoe* hibernates by entering larval diapause in the third or fourth instar, and larvae that were lighter than 5 mg had a small chance to survive ( Fischer and Fiedler 2002 ).

There were significant differences in development time and growth rate between direct developing and diapause developing pathways in *H. armigera* . Direct developing individuals had a significantly shorter larval development time and higher growth rate compared with diapause developing individuals. The differences may reflect the evolution of life history in this moth. In temperate areas, a large proportion of the year is unfavorable for growth and reproduction ( Danks 1994 ; Nylin et al. 1995 ). Therefore, for the direct developing pathway, a short development time is obviously beneficial for populations to make full use of the effective growth seasons and to extend their reproduction period. For the diapause developing pathway, because diapause for most insects is induced long before unfavorable environments, diapause individuals have enough time to grow to diapause stage and reach a critical pupal weight to overwinter successfully.

There is considerable variation in the degree of sexual size dimorphism within insect species. Females are usually larger than males, whereas only 7% of insect species with sexual size dimorphism data in the literature have larger males than females ( Table 1 in Stillwell et al. 2010 ). Interestingly, *H. armigera* shows male-biased sexual size dimorphism; the pupal and adult weights were significantly greater in males than females in both developmental pathways. Furthermore, female pupae lost more weight during metamorphosis compared with male pupae; thus, the significant sexual size dimorphism appeared at the adult stage. In *H. armigera* , females in both developmental pathways emerged earlier than males at all rearing temperatures ( Table 1 ), showing the protogyny phenomenon, and this is because female pupae develop faster than male pupae. The difference in pupal development time between females and males might be related to pupal weight because relatively small female pupae, which lost more weight during metamorphosis, indicated relatively higher metabolic rate, thus resulting in the early emergence of females. In the butterflies *Pararge aegeria* and *L. tityrus* , females with higher weights present the protandry phenomenon, in which male pupae lost more weight during metamorphosis and emerged earlier ( Gotthard et al. 1994 ; Fischer and Fiedler 2000 , 2001). Therefore, the pupal weight might determine emergence pattern (protandry or protogyny). The biological reason for this emergence pattern is not well-understood in insects ( Thornhill and Alcock 1983 ), but it has been demonstrated in other lepidopteran species and could represent an evolutionary strategy to promote mating between individuals from distinct populations ( Uematsu and Morikava 1997 ; Bento et al. 2006 ). It is also possible that this emergence pattern makes males to contribute nutrient-rich spermatophores to their partners, meaning male size might have a direct benefit on offspring number or quality.

Diapause is generally believed to be costly. A number of studies showed that diapause individuals have shorter adult longevity compared with non-diapause individuals, such as the sawflies *Neodiprion sertifer* and *N. swainei* ( Sullivan and Wallace 1967 ; Lyons 1970 ), the flesh fly *Sarcophaga bullata* (Denlinger 1981); the maize stalk borer *Busseola fusca* ( Gebre-Amlak 1989 ), the predatory mite *Amblyseius andersoni* ( Van Houten et al. 1991 ), the bruchid *Kytorhinus sharpianus* ( Ishihara and Shimada 1995 ), the spider mite *Tetranychus urticae* ( Kroon and Veenendaal 1998 ), the blow fly *Calliphora vicina* ( Saunders 2000 ), and the parasitoid *Asobara tabida* ( Ellers and Van Alphen 2002 ). In the present study of *H. armigera* , the newly-emerged adults from diapausing individuals that experienced 76 days from hatching to pupation (45 days at 20° C, 31 days at 25° C for diapause termination) had significantly longer longevity under a 16:8 L:D photoperiod 25° C than the newly-emerged adults from direct developing individuals that experienced 49 days from hatching to pupation. This is a result of the larger body weight in diapause individuals because increased body mass is typically correlated with increased nutrient reserves, therefore larger body sizes in diapausing individuals are generally considered to be adaptive because of their greater reserves ( Hahn and Denlinger 2007 ) and may ameliorate the negative cost of diapause on postdiapause longevity.

Overall, the results reveal the different life-history strategies between different developmental pathways and between sexes in *H. armigera* . Such differences can allow a fine-tuned response of individuals to seasonality and environmental stochasticity, which aids in the persistence of the population.

## References

[R1] Bento JMS Nava D.E. Chagas MCM Costa AH Libardi DJ Parra JRP . 2006 . Biology and mating behavior of the coconut moth *Atheloca subrufella* (Lepidoptera: Phycitidae) . Florida Entomologist 89 : 199 – 203 .

[R2] Berner D Blanckenhorn WU . 2006 . Grasshopper ontogeny in relation to time constraints: adaptive divergence and stasis . Journal of Animal Ecology 75 : 130 – 139 . 16903050 10.1111/j.1365-2656.2005.01028.x

[R3] Bidau CJ Martí DA. 2007 . *Dichroplus vittatus* (Orthoptera: Acrididae) follows the converse to Bergmann's rule although male morphological variability increases with latitude . Bulletin of Entomological Research 97 : 69 – 79 . 17298684 10.1017/S0007485307004749

[R4] Blanckenhorn WU Fairbairn DJ . 1995 . Life history adaptation along a latitudinal cline in the water strider *Aquarius remigis* (Heteroptera: Gerridae) . Journal of Evolutionary Biology 8 : 21 – 41 .

[R5] Blanckenhorn WU Preziosi RF Fairbairn DJ . 1995 . Time and energy constraints and the evolution of sexual size dimorphism—to eat or to mate? Evolutionary Ecology 9 : 369 – 381 .

[R6] Chen C Zhou HY Xia QW Chen YS Xue FS . 2012 . Temperature-dependent development and protogyny in *Helicoverpa armigera* . Chinese Journal of Applied Entomology 49 : 867 – 873 .

[R7] Cullen JM Browning TO . 1978 . The influence of photoperiod and temperature on the induction of diapause in pupae of *Heliothis punctigera* . Journal of Insect Physiology 24 : 595 – 601 .

[R8] Danks HV . 1994 . Diversity and integration of life-cycle controls in insects. In: Danks HV, Editor . Insect Life-Cycle Polymorphism: Theory, Evolution and Ecological Consequences for Seasonality and Diapause Control . pp. 5 – 40 . Kluwer Academic .

[R9] Denlinger DL . 1981 . Basis for a skewed sex ratio in diapause-destined flesh flies . Evolution 35 : 1247 – 1248 . 28563396 10.1111/j.1558-5646.1981.tb04993.x

[R10] Dingle H . 1992 . Food level reaction norms in size‐selected milkweed bugs ( *Oncopeltus fasciatus* ) . Ecological Entomology 17 : 121 – 126 .

[R11] Ellers J Van Alphen JJM . 2002 . A tradeoff between diapause duration and fitness in female parasitoids . Ecological Entomology 27 : 279 – 284 .

[R12] Fischer K Fiedler K . 2000 . Sex-related differences in reaction norms in the butterfly *Lycaena tityrus* (Lepidoptera: Lycaenidae) . Oikos 90 : 372 – 380 .

[R13] Fischer K Fiedler K . 2001 . Sexual differences in life-history traits in the butterfly *Lycaena tityrus* : a comparison between direct and diapause development . Entomologia Experimentalis et Applicata 100 : 325 – 330 .

[R14] Fischer K Fiedler K . 2002 . Life-history plasticity in the butterfly *Lycaena hippothoe* : local adaptations and tradeoffs . Biological Journal of the Linnean Society 75 : 173 – 185 .

[R15] Gebre-Amlak A. 1989 . Phenology and fecundity of maize stalk borer *Busseola fusca* (Fuller) in Awassa, southern Ethiopia . Insect Science and its Application 10 : 131 – 137 .

[R16] Gotthard K Nylin S Wiklund C . 1994 . Adaptive variation in growth rate: life history costs and consequences in the speckled wood butterfly, *Pararge aegeria* . Oecologia 99 : 281 – 289 . 28313882 10.1007/BF00627740

[R17] Hahn DA Denlinger DL . 2007 . Meeting the energetic demands of insect diapause: Nutrient storage and utilization . Journal of Insect Physiology 53 : 760 – 773 . 17532002 10.1016/j.jinsphys.2007.03.018

[R18] Honěk A. 1993 . Intraspecific variation in body size and fecundity in insects: a general relationship . Oikos 66 : 483 – 492 .

[R19] Ishihara M Shimada M . 1995 . Tradeoff in allocation of metabolic reserves: effects of diapause on egg production and adult longevity in a multivotine bruchid, *Kytorhinus sharpianus* . Functional Ecology 9 : 618 – 624 .

[R20] Janz N Nylin S Wedell N . 1994 . Host plant utilization in the comma butterfly: sources of variation and evolutionary implications . Oecologia 99 : 132 – 140 . 28313958 10.1007/BF00317093

[R21] Kivelä S Välimäki P Mäenpää M. 2012 . Genetic and phenotypic variation in juvenile development in relation to temperature and developmental pathway in a geometrid moth . Journal of Evolutionary Biology 25 : 881 – 891 . 22356649 10.1111/j.1420-9101.2012.02478.x

[R22] Kroon A Veenendaal R . 1998 . Tradeoff between diapause and other life-history traits in the spider mite *Tetranychus urticae* . Ecological Entomology 23 : 298 – 304 .

[R23] Landman W Oudman L Duijm M . 1989 . Allozymic and morphological variation in *Ephippiger terrestris* (Yersin, 1854) (Insecta, Orthoptera, Tettigonioidea) . Tijdschrift voor Entomologie 132 : 183 – 198 .

[R24] Lyons LA . 1970 . Some population features of reproductive capacity in *Neodiprion swainei* (Hymenoptera: Diprionidae) . The Canadian Entomologist 102 : 68 – 84 .

[R25] Masaki S . 1967 . Geographic variation and climatic adaptation in a field cricket (Orthoptera: Gryllidae) . Evolution 21 : 725 – 741 . 28563073 10.1111/j.1558-5646.1967.tb03430.x

[R26] McGregor R . 1996 . Phenotypic selection by parasitoids on the timing of life history in a leafmining moth . Evolution 50 : 1579 – 1584 . 28565693 10.1111/j.1558-5646.1996.tb03930.x

[R27] Mousseau TA Roff DA . 1989 . Adaptation to seasonality in a cricket: patterns of phenotypic and genotypic variation in body size and diapause expression along a cline in season length . Evolution 43 : 1483 – 1496 . 28564251 10.1111/j.1558-5646.1989.tb02598.x

[R28] Nylin S . 1992 . Seasonal plasticity in life history traits: growth and development in *Polygonia c-album* (Lepidoptera: Nymphalidae) . Biological Journal of the Linnean Society 47 : 301 – 323 .

[R29] Nylin S Gotthard K . 1998 . Plasticity in life-history traits . Annual Review of Entomology 43 : 63 – 83 . 10.1146/annurev.ento.43.1.63 9444750

[R30] Nylin S Wickman PERO Wiklund C . 1995 . Life-cycle regulation and life history plasticity in the speckled wood butterfly: are reaction norms predictable? Biological Journal of the Linnean Society 55 : 143 – 157 .

[R31] Ohgushi T . 1996 . Consequences of adult size for survival and reproductive performance in a herbivorous ladybird beetle . Ecological Entomology 21 : 47 – 55 .

[R32] Pöykkö H Hyvärinen M. 2012 . To grow fast or to grow big? Time-limited larvae of Eilema depressum speed up their growth and reduce number of instars . Entomologia Experimentalis et Applicata 142 : 145 – 152 .

[R33] Peters RH . 1986 . The ecological implications of body size . Cambridge University Press .

[R34] Roff DA . 1992 . Evolution of life histories: theory and analysis . Chapman & Hall .

[R35] Roff DA . 2002 . Life History Evolution . Sinauer Associates .

[R36] Roff DA Mousseau T . 2005 . The evolution of the phenotypic covariance matrix: evidence for selection and drift in *Melanoplus* . Journal of Evolutionary Biology 18 : 1104 – 1114 . 16033584 10.1111/j.1420-9101.2005.00862.x

[R37] Saunders DS . 2000 . Larval diapause duration and fat metabolism in three geographical strains of the blow fly, *Calliphora vicina* . Journal of Insect Physiology 46 : 509 – 517 . 12770215 10.1016/s0022-1910(99)00137-7

[R38] Sibly R Calow P . 1986 . Why breeding earlier is always worthwhile . Journal of Theoretical Biology 123 : 311 – 319 .

[R39] Stearns SC . 1992 . The evolution of life histories . Oxford University Press .

[R40] Stillwell RC Blanckenhorn WU Teder T Davidowitz G Fox CW . 2010 . Sex differences in phenotypic plasticity affect variation in sexual size dimorphism in insects: from physiology to evolution . Annual Review of Entomology 55 : 227 – 245 . 10.1146/annurev-ento-112408-085500 PMC4760685 19728836

[R41] Sullivan CR Wallace DR . 1967 . Interaction of temperature and photoperiod in the induction of prolonged diapause in *Neodiprion sertifer* . The Canadian Entomologist 99 : 834 – 850 .

[R42] Thompson JN . 1988 . Evolutionary ecology of the relationship between oviposition preference and performance of offspring in phytophagous insects . Entomologia Experimentalis et Applicata 47 : 3 – 14 .

[R43] Thornhill R Alcock J . 1983 . The evolution of insect mating systems . Harvard University Press .

[R44] Uematsu H Morikava R . 1997 . Protogyny in diamondback moth, *Plutella xylostella* (Lepidoptera: Yponomeutidae) . Japanese Journal of Applied Entomology and Zoology 41 : 217 – 223 .

[R45] Van Houten YM Bruin J Veerman A. 1991 . Repeated induction and termination of diapause in the predacious mite, *Amblyseius potentillae* (Garman) (Phytoseiidae). In: Schuster R, Murphy PW, Editors . The Acari Reproduction, Development and Life-History Strategies . pp. 267 – 275 . Chapman & Hall .

[R46] Wei XT Zhou YC Xiao HJ Wang XP Bao ZM Xue FS . 2010 . Relationship between the natural duration of diapause and postdiapause reproduction in the cabbage beetle, *Colaphellus bowringi* (Coleoptera: Chrysomelidae) . European Journal of Entomology 107 : 337 – 340 .

[R47] Wickman PO . 1992 . Mating systems of *Coenonympha* butterflies in relation to longevity . Animal Behaviour 44 : 141 – 148 .

[R48] Wiklund C Friberg M . 2011 . Seasonal development and variation in abundance among four annual flight periods in a butterfly: a 20-year study of the speckled wood ( *Pararge aegeria* ) . Biological Journal of the Linnean Society 102 : 635 – 649 .

[R49] Wiklund C Nylin S Forsberg J . 1991 . Sex-related variation in growth rate as a result of selection for large size and protandry in a bivoltine butterfly, *Pieris napi* . Oikos 60 : 241 – 250 .

[R50] Wu KM Gong PY . 1997 . A new and practical artificial diet for the cotton bollworm . Insect Science 4 : 277 – 282 .

[R51] Wu KM Guo YY . 2007 . Geotype differentiation and regional migratory regularity of *Helicoverpa armigera* in China . Plant Protection 33 : 6 – 11 . (in Chinese)

[R52] Yela JL Herrera CM . 1993 . Seasonality and life cycles of woody plant-feeding noctuid moths (Lepidoptera: Noctuidae) in Mediterranean habitats . Ecological Entomology 18 : 259 – 269 .

